# Strengthening Israel’s neonatal intensive care nursing workforce

**DOI:** 10.1186/s13584-015-0020-0

**Published:** 2015-05-15

**Authors:** Sunny G. Hallowell, Barbara Medoff-Cooper

**Affiliations:** Center for Health Outcomes and Policy Research, University of Pennsylvania School of Nursing, 418 Curie Blvd, Philadelphia, PA 19104-4217 USA; University of Pennsylvania School of Nursing, Room 483 Fagin Hall, 418 Curie Blvd, Philadelphia Pennsylvania, 19104-4217 USA

## Abstract

Israel is experiencing a shortage of both physicians and nurses in a number of specialties, including neonatal intensive care. Inadequate hospital staffing and high patient demand contribute to the blurring of professional scope of practice boundaries between nurses and physicians. Striking similarities exist between the situation in Israel and the health services landscape in the United States more than three decades ago. This commentary explores changes related to nursing education, scope of practice legislation and hospital staffing learned through the U.S. experience that have the potential to inform health workforce changes in Israel through better nursing care.

Across Israel a national shortage of medical staff in the neonatal intensive care units (NICU) has required nurses to perform activities beyond their scope of practice [1]. Toren *et al.* identify several potential solutions to this crisis in the provision of care: (1) The need for clearer definition and understanding of the professional role of the nurse and the scope of practice of nursing; (2) Removal of organizational barriers that prevent nurses from practicing at their highest levels of education and clinical preparation; (3) Development of a team-based model of care; and (4) Development of an advanced level of nursing practice [1].

A significant challenge to the advancement of the nursing profession remains the negotiation of professional boundaries between nurses and physicians. Traditionally, this type of role transition occurs over time, as one group assumes a new role that the other relinquishes [1]. However, the ever-increasing birth rate and survival of preterm newborns due to medical and technical innovation has increased the demand for special-care neonatal units, generating an urgent and immediate need for change. In order to make infant care in the NICU more efficient, a shift in understanding and the development of a consistent model of care needs to be translated through educational institutions and national policy.

## Nurse education and professionalism in America

The conditions that preceded the development of the Advance Practice Registered Nurse (APRN) role in the U.S. (an increased demand for hospital care, higher medical complexity of patients and a shortage of physicians and nurses) are similar to the conditions currently experienced in Israel. A crucial educational shift occurred when the Bachelors of Science in Nursing (BSN) degree replaced the associate’s degree as the foundation of the educational trajectory for professional nursing.

From the turn of the century until the mid-1960’s, U.S. nurses were trained in hospitals where the diploma in nursing permitted entry to work in hospital settings [2]. Forward thinking nursing leaders believed the future of the profession depended on moving nurse training into higher education which resulted in the phasing out of most hospital-based diploma programs and implementation of the college level Associate Degree in Nursing (ADN) [2]. The technically focused two-year training of the ADN became highly popular and programs expanded rapidly across the nation.

By the time the American Nurses Association published the 1965 position paper calling for the Baccalaureate in Nursing Science (BSN) degree to become the minimum requirement for entry into professional nursing practice, a three-tiered system of nursing education had been established in the U.S. [2]. This ignited an impassioned debate to distinguish the merits of technical nurse (ADN prepared) and professional nurse (BSN prepared) training, since both groups were eligible to enter practice by passing the same National Board Certification Exam to assume the title Registered Nurse (RN) [2]. The BSN degree prepared nurses to move from task-based proficiencies emphasized in the ADN curriculum to higher-level competencies to provide a foundation for knowledge, theory and critical thinking skills [3]. Furthermore, competencies in decision making, quality improvement, systems thinking, and team leadership became part of every nurse’s professional formation [3].

Israel already requires educational training equivalent to a 4-year BSN degree as the minimal educational standard for entry into professional nursing practice. Many countries including the U.S., U.K., Germany, Spain and others continue to struggle to establish this educational model. Maintaining the BSN degree as the foundation of entry into professional practice is critical for the future development of advanced roles for nurses and establishing curriculum for advanced degrees at the Master’s and Doctoral levels.

## Development of the Advance Practice Nurse (APRN) role for neonatal intensive care in the US

As the healthcare landscape in U.S. continued to change, by the 1970’s nurses began to specialize within patient populations and care became both disease and population specific. Whereas sick infants were typically cared for in pediatric wards, medical and technical advancement allowed for greater survival of sick and premature infants [4]. In the Neonatal Intensive Care Unit (NICU) day-to-day medical care depended on the independent practice of nurses to perform tasks such as intravenous (IV) blood draws, traditionally under the purview of the physician [4]. The collaborative relationship between physicians and nurses culminated in 1973 with the Blue Ribbon Commission, funded by the March of Dimes. The resulting document from that pivotal meeting set the training standard endorsed by the American Nurses Association for Neonatal Nurse Practitioners (NNPs) for the next two decades [4].

Pioneering nurse educators and researchers lead the conceptualization and development of the content, methods and implementation of the Advance Practice Nurse (APRN) educational curriculum. In the U.S. NNPs are exclusively trained with the Master of Science in Nursing (MSN) and/or more recently with the Doctor of Nursing Practice (DNP) degree as well. The DNP has quickly become the terminal degree for all areas of advanced nursing practice and is designed to provide education for advanced nursing practice roles, which include those focused on practice at the aggregate, systems, or organizational level [5]. Presently, APRN roles are divided into four categories: Nurse Anesthetist, Nurse-Midwife, Clinical Nurse Specialist and Nurse Practitioner (NP), within six population foci: Family, Adult-Gerontology, Neonatal, Pediatrics, Women’s Health, Psychiatric-Mental Health [6]. The NNP is a subspecialty APRN role where educational curriculum and clinical experiences are specific to the neonatal patient population [6]. Graduates may practice in any state; however, individual states determine scope of practice legislation. To date, 19 states and the District of Columbia offer NPs full practice authority (without a collaborative agreement with a physician), increasing access to healthcare for many Americans.

An important difference between the models of nursing education in the U.S. and Israel is that, in the U.S., educational preparation necessary to enter the profession of nursing is designed and developed by nursing educators in universities rather than state or national governmental agencies [3]. Few universities in Israel offer a Master’s level degree in Nursing. Development of an evidence-based clinical curriculum would be necessary for APRN training. The Post Basic Education (PBE) option provided by the Ministry of Health may provide a helpful foundation for developing this clinical curriculum.

## Creating a health care team

Despite divergent training and philosophies, nurses and physicians share a common goal - to improve and provide benevolent, safe, high-quality patient care. The 2001 Institute of Medicine (IOM) report Crossing the Quality Chasm revolutionized how hospitals and policy makers viewed the structure of the health care system and highlighted the inefficient use of resources [7]. Key recommendations called for all health care organizations and professional groups to promote health care that is safe, effective, client-centered, timely, efficient, and equitable [7]. During the same period, a breakthrough study associated significant medical errors with sleep deprivation experienced by medical interns which resulted in the Accreditation Council for Graduate Medical Education (ACGME) limiting the work-hours of interns to 80 h weekly [8]. The growing supply of nurse practitioners filled patient care needs created by the necessity for safe, effective and high quality health care and the reduction in physician work hours.

The evolution of Neonatology as a specialty required the re-negotiation of traditional inter-professional boundaries by both physicians and nurses. This critical shift in thinking was necessary to operationalize rapidly developing clinical processes implemented to improve infant care. Although the re-negotiation of skills occurred over time, the APRN role evolved to allow NNPs to take the lead of the delivery room team, fulfilling tasks traditionally performed by a physician (*i.e.* infant resuscitation, central line insertion) and working collaboratively with physicians, respiratory therapists, medical interns and nurses [3, 4]. The level of autonomy required for NNPs to shift into the role of leadership in the NICU and to practice to the full extent of their education and training embodied the goals of another landmark IOM report [3]. The Future of Nursing: Leading Change, Advancing Health provided the necessary impetus to inspire the level of collaboration necessary between nurses, physicians and hospitals: (1) Nurses should practice to the full extent of their education and training; (2) Nurses should achieve higher levels of education and training through an improved education system that promotes seamless academic progression; (3) Nurses should be full partners, with physicians and other health care professionals, in redesigning health care in the U.S. [3].

The design of team-based care in the NICU is not limited to nurses and physicians; in many settings the team is comprised of registered dieticians, social workers, NNPs, physician’s assistants (PAs), lactation consultants, physical/occupational therapists, parents and families of the infants. This multi-disciplinary team approach may not be present in NICUs in Israel but is common in the U.S. and is a feature critical to good decision-making and enhances both physician and nursing care. Yet, the struggle to achieve a team-based approach in the U.S. continues to challenge modern providers due to deep-rooted culture beliefs related to the physician being the leader of the healthcare team.

## Shaping the future of neonatal nursing in Israel

Translating the lessons from one country’s experience to another country is challenging due to differences in economic, social and cultural circumstances. Nonetheless, the use of international benchmarks for nurse education, hospital work environments and staffing may help policy makers and legislators shape the future of nursing in Israel.

Evidence has shown that better NICU work environments and staffing have been associated with better patient safety, lower risks of mortality, blood stream infection, severe intraventricular hemorrhage and improved support for infant nutrition and breastfeeding [9–13]. In addition to improved patient outcomes, quality and safety of care, better hospital work environments and staffing ratios are modifiable factors that have been associated with alleviating nursing shortages and attracting candidates into the profession of nursing [14]. Responses to nurse staffing shortages by increasing the number of licensed practical nurses may be a short-term staffing solution, but this is not the answer to long-term patient care needs or improved patient outcomes [14, 15].

Over a decade of national and more recently, international research has associated higher nurse education with improved patient outcomes [16–20]. Studies have shown that a 10 % increase of baccalaureate prepared nursing staff has been associated with reduction in mortality within 30 days of admission for adults admitted to this hospital for common surgical procedures and for post-surgical complications [16–20]. The association between higher nurse education and improved patient outcomes may be related to better critical thinking and clinical judgment skills associated with BSN preparation [18]. Administrative decisions focused on increasing the production and employment of baccalaureate prepared nurses may also improve these outcomes in hospitals in Israel. This work has not yet been replicated in the neonatal literature but is an area for future investigation.

Greater public awareness of the profession of nursing and financial investments by universities, hospitals and government agencies has increased the profile of nursing as a career [3]. Israel may benefit from similar investments in its own domestically trained nurses [14].

In the U.S., nurses are the most trusted health care professionals [21]. Candidates choose the profession to gain the knowledge and skill of working with patients; the opportunities to specialize and become a leader in all areas of healthcare; the challenge of lifelong learning; the collaborative nature of working in a team and the flexibility of schedules [22]. Another reason for the success of the nursing model in the U.S. is the promise of financial reward for higher education. The pay differential attracts nurses to pursue higher education, accept the increased responsibility in patient care and develop a sense of professional leadership. This allows nurses to see the value in the investment in their education but also allows physicians to understand the value of hiring highly skilled nurses into practice, and measured by improved patient outcomes, satisfaction and quality of care.

The demand for patient care in Israel has increased the need for a more qualified health care workforce. U.S. studies have repeatedly demonstrated improved patient outcomes and satisfaction with investments in building hospital work environments that employ nurses with higher levels of education and ensure stable nurse-to-patient staffing ratios [14, 15]. To reinforce development of a competent and efficient nursing workforce of the healthcare workforce, consistent implementation of scope of practice regulations across institutional settings is necessary to operationalize desirable features of the hospital work environment. In the NICU improved communication between nurses and physicians is a fundamental requirement to facilitate better management of infant care. Building a NICU environment that has robust understanding, willingness and capacity to develop an interdisciplinary team approach to patient care would reinforce critical-decision making for both nurses and physicians necessary to provide much needed care to Israel’s sick infants and their families. An important feature of modern health care is expansion of the nursing role to an advanced level of clinical practice. In order to successfully implement the APRN role, educational and legislative support for professional nurses at all levels of training must be established. The role of the APRN emerging in Israel holds the promise for better access to patient care.
